# Occlusal Assessment and Treatment Planning Under General Anaesthesia in a Patient With Complex Needs: A Case Report and Reflective Analysis

**DOI:** 10.1111/scd.70282

**Published:** 2026-10-23

**Authors:** Naomi Murray, Andrew Geddis‐Regan

**Affiliations:** ^1^ University Dental Hospital of Manchester Manchester University NHS Foundation Trust Manchester UK; ^2^ Division of Dentistry University of Manchester Manchester UK

**Keywords:** consent, capacity, general anaesthesia, occlusal trauma

## Abstract

**Background:**

Occlusal assessment under general anaesthesia (GA) is inherently limited, as functional jaw movements and dynamic occlusal loading cannot be replicated intraoperatively. In patients who require GA as the only feasible treatment modality, this may pose challenges for dental professionals planning care intra‐operatively.

**Case Report:**

A 37‐year‐old male with autism, severe learning disability, and epilepsy underwent extraction of LR6, LR7, and LR8 under GA for gross caries, alongside professional mechanical plaque removal. Just over one year later he presented with self‐injurious behaviors attributable to dental pain. His LR5 was grade 3 mobile, with an OPG demonstrating 50% horizontal and near‐complete vertical bone loss; prior imaging had shown healthy bone levels. Primary occlusal trauma was the working diagnosis, with LR5 having assumed the entire posterior occlusal load following molar extraction. Extraction under local anaesthesia was not tolerated; a further best interests decision was reached and LR5 was removed under GA with concurrent socket biopsy, which excluded malignancy.

**Outcome:**

At six‐month follow‐up the patient remained asymptomatic with no recurrence of self‐injurious behaviors, and imaging demonstrated slow but progressive bone fill of the socket.

**Discussion:**

This case illustrates the inherent difficulty of predicting real‐world occlusal function when GA is the only treatment modality available, and the ethical and clinical complexity of best interests decision‐making in this patient group.

## 1 Introduction

The practice of Special Care Dentistry (SCD) is concerned with providing oral health care for adolescents and adults (defined as 16 years old and above) with impairments that limit their ability to access routine dental care, including those with learning disabilities [1]. People with a learning disability have poorer health and experiences of healthcare than their non‐disabled peers, as challenges with communication, understanding, behavior, and/or reliance on their care teams can lead to illnesses going undiagnosed [2]. In addition, they face significant barriers to accessing standard dental care, including individual‐level factors, such as difficulty tolerating treatment, and organizational‐level factors, such as a shortage of dental professionals with the requisite knowledge, training and confidence in caring for people with disabilities [3, 4].

The use of general anaesthesia (GA) is therefore an essential tool in the provision of SCD; it helps overcome difficulties in obtaining sufficient patient cooperation, particularly in individuals with significant behavioral challenges or cognitive impairments that preclude them from understanding the need for dental care [5]. Furthermore, GA enables comprehensive dental treatment to be completed in a single episode and, in some cases, may constitute a less restrictive option, particularly when compared with alternative treatment modalities that necessitate multiple complex visits, which can impose significant logistical and emotional burdens.

Although the use of GA has become considerably safer since the turn of the 21st century [6], it continues to carry a higher level of risk compared with other treatment modalities, such as local anaesthesia or conscious sedation; therefore, its use requires careful and judicious consideration [5]. In the United Kingdom, established guidance informs its use in SCD, supporting a comprehensive approach to case selection, assessment and planning [7]. Intra‐operative treatment planning and delivery may differ from that which is provided by other forms of Dental Behavior Support; definitive and predictable techniques should be prioritized to minimize the risk of post‐operative complications warranting further intervention, such as loss of vitality following deep restorations [7]. Treatment planning must also take into account factors including, but not limited to, the patient's caries risk and ability to cooperate with oral hygiene and simple dental care. Consequently, these considerations may justify a more radical treatment plan when providing care under GA [7].

With the objective of achieving a stable, symptom‐free dentition that supports masticatory function, the post‐operative occlusal state must be carefully considered and planned to ensure optimal stability and function [7]. The dental team should endeavor to assess occlusion intra‐operatively, addressing any occlusal interferences introduced during restorative treatment, and remain mindful of situations in which post‐operative occlusal adjustment may not be feasible due to limitations in patient cooperation. However, intra‐operative occlusal assessment may not always be achievable, particularly in cases where a nasal endotracheal tube is not used. This case report highlights the challenges that arise when dental care can only be provided under GA, the limitations this places on occlusal assessment and treatment planning, and the importance of reflective clinical practice.

## 2 Case Report

### 2.1 Patient History

A 37‐year‐old male was referred to a University Dental Hospital Department of SCD for dental treatment under GA. The referral originated from the Community Dental Service due to a history of ongoing pain from the lower right quadrant. The patient had limited verbal communication but was able to indicate pain by pointing to the affected area.

His medical history included autism spectrum disorder, severe learning disability, and well‐controlled epilepsy. He was prescribed a combination of anti‐epileptic and antipsychotic medicines. He lived in 24‐hour supported residential care, though his mother remained closely involved in his care. He required two‐to‐one support due to behaviors that challenge, which had increased in frequency since the onset of his dental pain. Regarding oral hygiene, he allowed carers to brush his teeth twice daily; however, his diet was high in sugar, including frequent consumption of fizzy drinks.

No formal dental records were available for this patient as an adult. His mother reported that he had received dental treatment under GA as a child, and restorations visible on the initial orthopantomogram (Figure 1) are consistent with this history.

**FIGURE 1:**
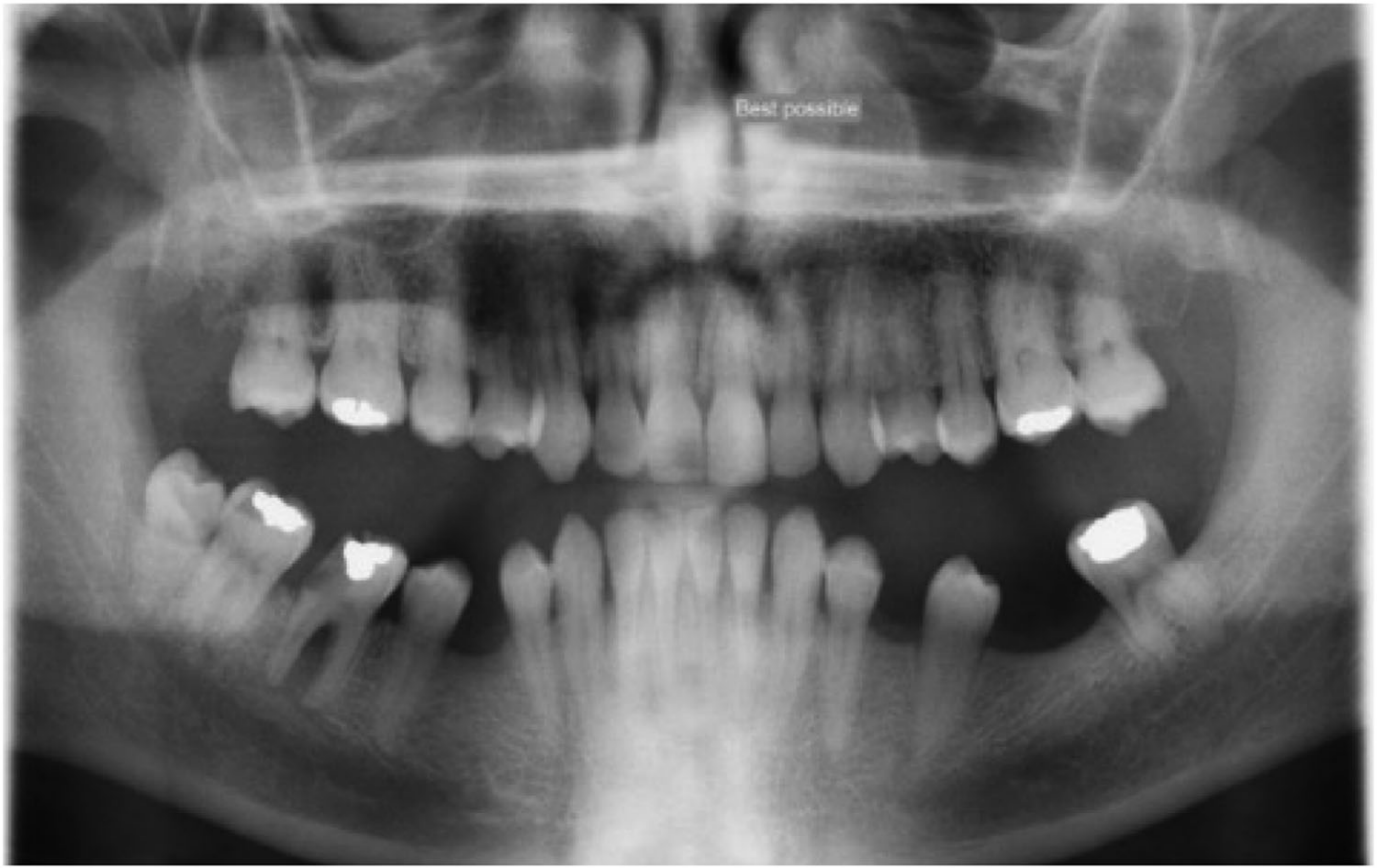
Orthopantomogram at initial presentation demonstrating gross caries LR6 and LR7, with a partially erupted LR8.

Clinical examination was challenging due to limited patient cooperation; however, he allowed a visual examination using a plastic mirror, and gross caries was identified on the lower right first and second molars, along with a partially erupted lower right third molar. LR7 demonstrated a lingual fracture with subgingival caries at the fracture site, rendering it unrestorable: extraction was therefore indicated. Despite difficulties with examination, the patient was able to tolerate an orthopantomogram (OPG), which confirmed the clinical findings (Figure 1)

Given the patient's significant learning disability , he was assessed as lacking capacity to consent under the UK Mental Capacity Act, 2005 [8]. A Best Interests Meeting was conducted with his mother and care team, and it was agreed that dental treatment under GA was in his best interest. The patient subsequently underwent extraction of LR8, LR7 and LR6, and professional mechanical plaque removal under GA. A nasal endotracheal tube was used, permitting intraoperative static occlusal assessment, though this does not replicate dynamic functional occlusion. Pre‐operative investigations including venepuncture, ECG, and other routine assessments were not obtainable due to the patient's inability to cooperate. Anaesthesia risk assessment followed established departmental protocols, with the anaesthetist reviewing the patient physically on the day of the procedure and modifying the anaesthetic plan accordingly. The procedure was uneventful and the patient was discharged to the care of his residential support team on the day.

### 2.2 Presenting Problem

Just over one year later, the care team contacted the department, reporting that the patient had a broken tooth in the lower right quadrant. They requested an urgent review, as he appeared to be in significant pain and had begun exhibiting self‐injurious behaviors, including hitting his head and biting his hands and arms. There was no history of trauma or seizure activity to explain a fractured tooth.

The patient was assessed the following day. Clinical examination revealed that tooth LR5 was intact but demonstrated grade 3 mobility. A new OPG showed approximately 50% horizontal bone loss, complete vertical bone loss distal and 90% vertical bone loss mesial to LR5 (Figure 2). Differential diagnoses of primary occlusal trauma, vertical root fracture, and bony or soft‐tissue pathology were considered. Iatrogenic trauma during the preceding extractions was noted as a theoretical possibility, though the degree and localized pattern of bone loss would be atypical for this mechanism. The rapidity and severity of the bone loss, specifically near‐complete vertical loss within just over one year in a tooth with previously healthy radiographic bone levels, was noted as atypical and clinically significant.

**FIGURE 2:**
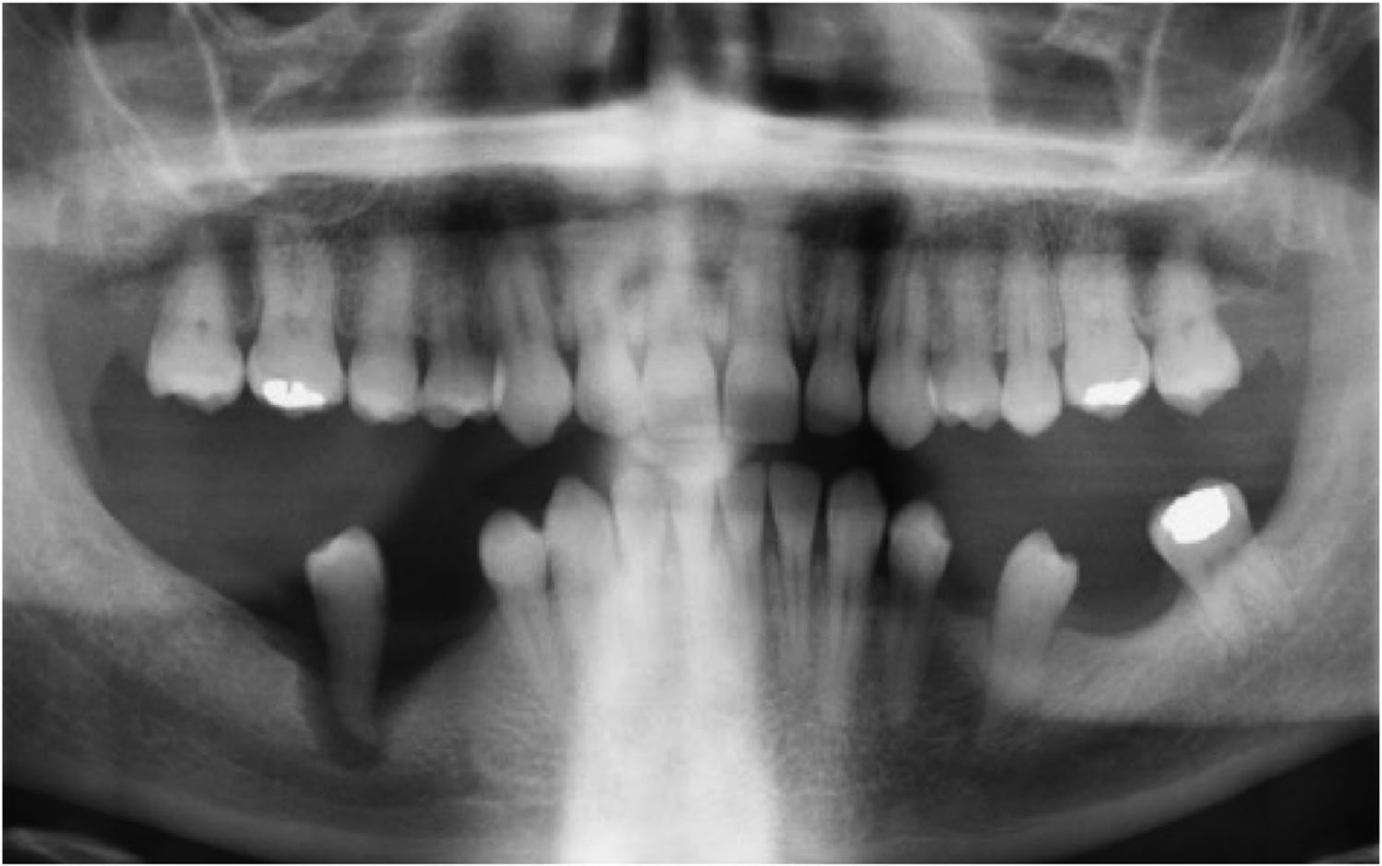
Orthopantomogram showing approximately 50% horizontal bone loss and complete vertical bone loss both distal and 90% vertical bone loss mesial to LR5.

### 2.3 Management

An attempt was made to extract the LR5 at the assessment appointment due to the urgency of care required. However, local anaesthesia could not be administered safely, and the procedure was therefore abandoned. In light of the patient's complex needs and the urgency of treatment, a further best interests meeting was convened, and it was agreed that removal of LR5 under GA was in the patient's best interest.

Although GA may be considered a restrictive intervention for a relatively limited treatment requirement, alternative management options were carefully evaluated. Dentist‐led conscious sedation (using midazolam) was considered; however, concerns were raised regarding the patient's ability to tolerate dental care even with this form of support. During a previous GA, substantial midazolam premedication had been required to facilitate inhalational induction, suggesting that conscious sedation would be unlikely to provide an adequate level of cooperation and might instead exacerbate agitation and distress. Anaesthetist‐led sedation was also explored; however, this service was not routinely available within the organization. Consequently, its provision would have been logistically challenging due to the limited availability of anaesthetists comfortable with providing multi‐drug sedation for shared‐airway cases. Given the urgency of treatment, these practical constraints further reduced the feasibility of this option. Following multidisciplinary discussion, the treating anaesthetist considered GA to represent the safest and most predictable approach, taking into account the patient's behavioral presentation, premedication requirements, and physical stature.

Consequently, LR5 was removed under GA. An i‐gel supraglottic airway device was used for this second, more limited procedure. This simplified the anaesthetic process and expedited recovery. A biopsy of the extraction socket was also taken to exclude soft tissue or bony pathology as the cause of sudden tooth mobility. Histological examination revealed hyperplastic mucosa with chronic active inflammation and bacterial colonies, with no evidence of malignancy. Given the absence of visible cracks on the root surface of the extracted tooth, a working diagnosis of primary occlusal trauma was made.

The patient was reviewed in clinic six months later. He remained asymptomatic, with no recurrence of self‐injurious behavior. A follow‐up OPG demonstrated slow but progressive bone filling of the extraction socket (Figure 3).

**FIGURE 3:**
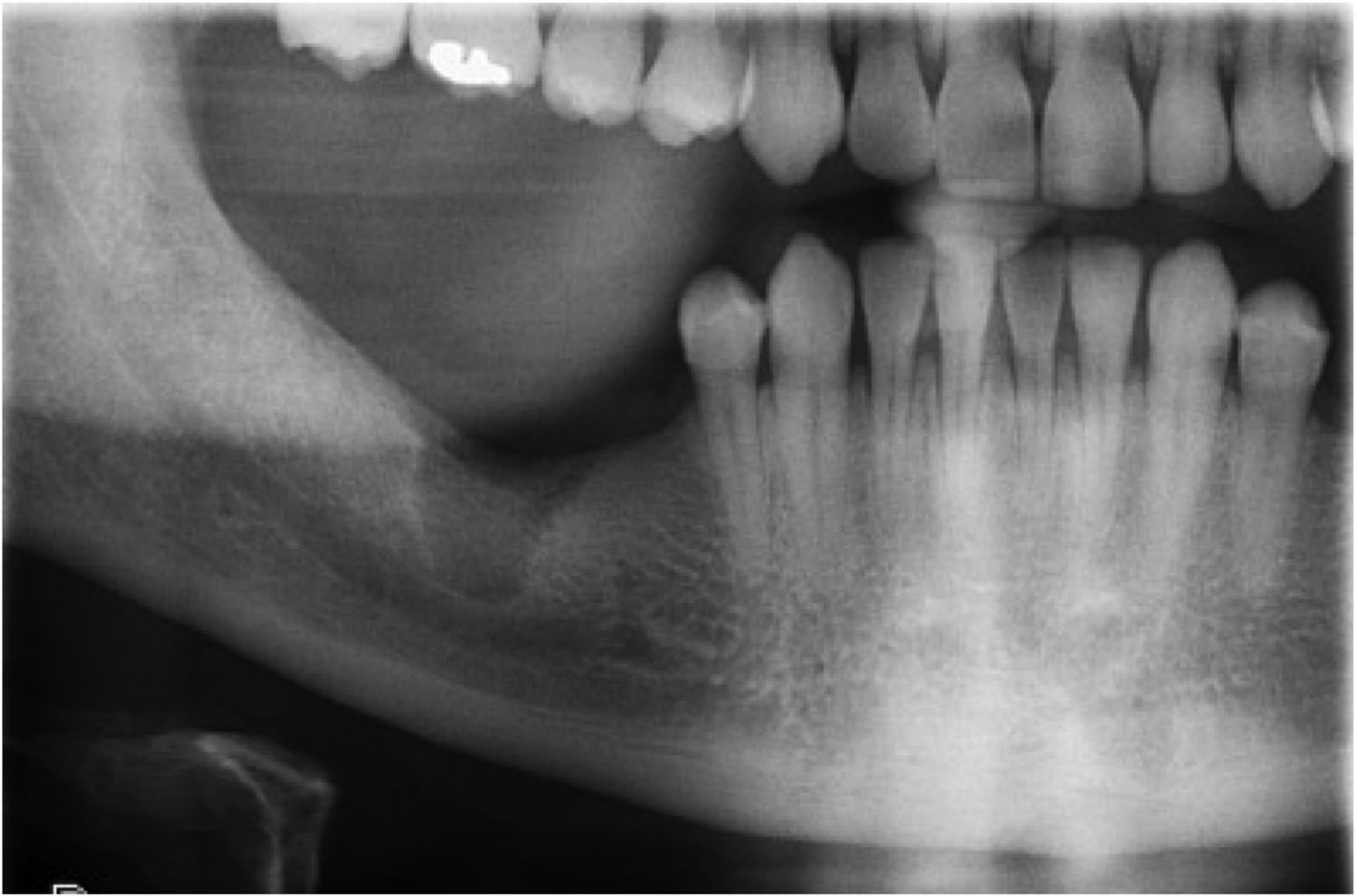
Follow‐up orthopantomogram six months after extraction of LR5, demonstrating progressive bone fill of the extraction socket.

## 3 Discussion

This case prompted critical reflection on two key considerations: whether the initial management approach was optimal, and whether alternative decisions may have resulted in a different clinical outcome. Reflective practice is essential in healthcare [9], particularly in cases where patients lack decision‐making capacity and clinicians are required to act in their best interests.

The primary working diagnosis in this case was primary occlusal trauma, which is defined as injury to the periodontium resulting from excessive occlusal forces exceeding the adaptive capacity of the supporting tissues [10]. Following the initial GA, extraction of the lower right molars resulted in LR5 bearing the entirety of the posterior occlusal load. This redistribution of occlusal forces is likely to have contributed to the subsequent deterioration observed. Figure 4 presents a schematic representation of the pathological process underlying occlusal trauma and the subsequent progression of pathological changes.

**FIGURE 4:**
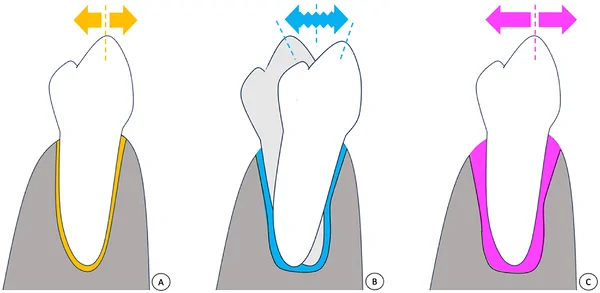
(A) Healthy periodontal ligament space, with normal physiological tooth movement. (B) Excessive loading or ‘jiggling’ forces causing the periodontal ligament space to widen. (C) Necrosis of the periodontal ligament and bone resorption permitting tooth mobility.

A vertical root fracture was also considered as a differential diagnosis; however, such fractures are more commonly associated with endodontically treated teeth [11], and no evidence of fracture was identified upon clinical inspection following extraction.

The patient's prescribed medicines are also relevant to the clinical picture. Several anti‐epileptic and antipsychotic agents are associated with xerostomia as a side effect, which in turn increases susceptibility to periodontal disease. While this may have contributed to background periodontal vulnerability, it does not account for the localized and rapid pattern of bone loss seen around LR5, which is more consistent with the primary occlusal trauma diagnosis.

This case also highlights the inherent challenges associated with assessing and establishing occlusion under GA. Clinical guidelines emphasize the importance of achieving a stable and functional occlusal outcome following treatment under GA [7]. However, several factors may compromise the accuracy of intraoperative occlusal assessment. These include the presence of an endotracheal tube, which, if orotracheal rather than nasotracheal, may preclude occlusal evaluation altogether; the use of a throat pack, which can displace the tongue anteriorly; and the altered positioning of oral soft tissues in the supine patient, compounded by the effects of muscle relaxants administered during anaesthesia [12]. Collectively, these factors limit the clinician's ability to reliably reproduce functional occlusion at the time of treatment.

Importantly, these limitations are not incidental to this case but a key part of it. For patients who can tolerate routine dental care with local anaesthesia, occlusal monitoring and adjustment across multiple visits remains possible. For a patient such as this, where GA represented the only feasible treatment modality, no such iterative approach was available. The clinical consequence of undetected occlusal trauma therefore could not be identified until it had already produced significant pathology. This distinguishes the learning point here from one that would apply regardless of the treatment modality used.

Beyond the clinical challenges of treatment planning under GA, this case also highlights the ethical and legal complexities associated with decision‐making for patients who lack capacity. In England and Wales, the Mental Capacity Act 2005 provides the legal framework governing decision‐making for individuals aged 16 years and over who are unable to make decisions for themselves [8]. Within this framework, it is essential that carers, family and/or advocates if needed are appropriately informed about the implications of treatment under GA as part of the best interests decision‐making process. This includes recognizing that treatment delivered under GA may, in some circumstances, be more extensive than would ordinarily be undertaken under local anaesthesia, reflecting the practical limitations of repeated interventions and the need to provide definitive care within a single episode.

Furthermore, this case underscores the delicate balance between establishing a stable occlusal scheme and avoiding the unnecessary extraction of teeth without clear pathology. While a more radical approach at the initial GA, such as removal of LR5, may have prevented the subsequent complication, it is equally plausible that such intervention could have resulted in undesirable consequences elsewhere in the dentition, including increased loading of the anterior teeth. This uncertainty highlights the difficulty of making definitive treatment decisions in complex cases, particularly when influenced by the limitations of GA being the only accepted treatment modality.

In retrospect, it is challenging to determine whether an alternative approach would have yielded a more favorable outcome, as such assessments are inherently subject to hindsight bias [13]. Nevertheless, this case provides valuable lessons for clinical practice. For example, should extraction of the lower left second molar (LL7) be required at a later stage (a plausible possibility given the extent of the existing restoration and its susceptibility to eventual failure), consideration could be given to the concurrent removal of the lower left second premolar, even in the absence of overt pathology, to achieve a more balanced distribution of occlusal forces and mitigate the risk of LL5 undergoing deterioration similar to that observed in the LR5. Although elective removal of LL5 may appear radical, several patient‐specific factors support a more preventative approach: the risk of self‐injurious behavior in response to even minor dental symptoms, the substantial logistical support each appointment required, the need for high‐dose premedication, and the fact that all treatment could only be provided under GA. Collectively, these factors would support consideration of elective extraction of LL5 to reduce the likelihood of future interventions, cumulative risk, patient distress, and healthcare resource utilization.

## 4 Conclusion

In conclusion, this case illustrates the challenges associated with predicting functional occlusal outcomes following treatment under GA, even when treatment is carefully planned and delivered in accordance with clinical guidelines. It emphasizes the importance of reflective practice in dentistry, encouraging clinicians to critically evaluate their decisions, learn from clinical outcomes, and apply these insights to future patient care.

While optimal outcomes cannot always be achieved in complex cases, each clinical experience provides an opportunity to refine decision‐making and enhance the quality of care delivered.

## Author Contributions

NM and AGR treated the patient in this case report. NM wrote the initial manuscript. NM and AGR critically appraised the manuscript.

## Ethics Statement

Ethical approval was not required for this case report.

## Consent

Formal consent was not required for this case report as no patient identifiable data was used.

## Conflicts of Interest

The authors declare no conflicts of interest.

## Data Availability

No datasets were generated or analyzed in this article. Due to patient confidentiality, no additional data is available. AI was used to proof‐read only, not to generate any content.
